# A randomized controlled trial comparing two vitrification methods versus slow-freezing for cryopreservation of human cleavage stage embryos

**DOI:** 10.1007/s10815-013-0145-4

**Published:** 2013-12-08

**Authors:** Giovanna Fasano, Nicolas Fontenelle, Anne-Sophie Vannin, Jamila Biramane, Fabienne Devreker, Yvon Englert, Anne Delbaere

**Affiliations:** 1 Research Laboratory on Human Reproduction, Faculty of Medicine, Université Libre de Bruxelles, Campus Erasme, Bruxelles, Belgium; 2 Fertility Clinic, Department of Obstetrics and Gynecology, Hôpital Erasme, Université Libre de Bruxelles, Route de Lennik, 808, 1070 Bruxelles, Belgium

**Keywords:** Human cleavage stage embryo, Vitrification, Slow freezing, Survival and cleavage rates, Clinical outcomes

## Abstract

**Purpose:**

To compare two different vitrification methods to slow freezing method for cryopreservation of human cleavage stage embryos. Design: Prospective randomised trial. Setting: University assisted reproduction centre. Patient(s): 568 patients (mean age 33.4 ± 5.2) from April 2009 to April 2011.

**Methods:**

1798 supernumerary good-quality cleavage stage embryos in 645 IVF cycles intended to be cryopreserved were randomly allocated to three groups: slow freezing, vitrification with the Irvine® method, vitrification with the Vitrolife® method. Main Outcome Measure(s): Embryo survival and cleavage rates, implantation rate.

**Results:**

A total of 1055 embryos were warmed, 836 (79.2 %) survived and 676 were finally transferred (64.1 %). Post-warming embryos survival rate was significantly higher after vitrification (Irvine: 89.4 %; Vitrolife: 87.6 %) than after slow freezing (63.8 %) (*p* < 0.001). No differences in survival rates were observed between the two vitrification methods, but a significant higher cleavage rate was observed using Irvine compared to Vitrolife method (*p* < 0.05). Implantation rate (IR) per embryo replaced and per embryo warmed were respectively 15.8 % (41/259) and 12.4 % (41/330) for Irvine, 17.0 % (40/235) and 12.1 % (40/330) for Vitrolife, 21.4 % (39/182) and 9.9 % (39/395) for slow-freezing (NS).

**Conclusions:**

Both vitrification methods (Irvine and Vitrolife) are more efficient than slow freezing for cryopreservation of human cleavage stage embryos in terms of post-warming survival rate. No significant difference in the implantation rate was observed between the three cryopreservation methods.

## Introduction

In vitro fertilization (IVF) cycles are often characterised by the production of excess human embryos which can be cryopreserved and used for the patients to achieve pregnancy in subsequent warming cycles [1]. Embryo cryopreservation improves the cumulative pregnancy rate per IVF cycle allowing additional chances of pregnancy without re-exposure to exogenous gonadotropins and subsequent oocyte retrieval procedure. Embryo cryopreservation allows to perform fresh elective single embryo transfer (SET), avoiding multiple gestations [2–5]. Embryo freezing is also used as strategy to prevent ovarian hyperstimulation syndrome [6–8] or to delay embryo transfer if endometrial preparation is not optimal [9, 10]. The first report of successful pregnancy from transferred cryopreserved human embryos was published 29 years ago [11]. Since then, cryopreservation became essential in assisted reproductive technologies to optimize the efficiency of IVF cycles [12–15]. Conventional slow-freezing protocols have been extensively used for cryopreservation of human embryos. These procedures are based on low cryoprotectant concentrations and a slow cooling rate. Vitrification is an increasingly popular method, based on an extreme viscosity of cryoprotectant solutions and a high cooling rate. The assessment of benefits/risks as well as cost/efficiency of the different cryopreservation methods has been recently reviewed [16–18]. Both procedures have advantages and limitations and at present, a wide variety of cryopreservation protocols of slow-freezing and vitrification are available. The main issue during embryo cryopreservation procedure, in both cooling and warming steps, are the chilling injury, the intracellular ice formation, and the fracture damages [19, 20]. To overcome them, all cryopreservation strategies are using different cryoprotectant solutions and cooling-warming rates. Vitrification has been extensively tested on human blastocyst stage embryos [21–23]. Vitrification has also been applied successfully for the cryopreservation of human cleavage stage embryos [24, 25]. It has to be noted that improved slow-freezing methods can produce comparable results [26, 27]. However, there are still only few randomized clinical trials addressing the optimal method of cryopreservation for human cleavage stage embryos [28–30].

The aim of this study was to compare the slow-freezing method usually performed in our Fertility Clinic for cryopreservation of human cleavage stage embryos to two different vitrification methods using commercially available media. High security devices were used for all procedures. Data analyzed included survival rate, cleavage rates of embryo after warming and clinical outcomes after transfer.

## Materials and methods

The study was approved by the Ethics Committee of Hospital and conducted at the Fertility Clinic from April 2009 to April 2011.

Before starting their IVF cycle, patients were thoroughly informed about the comparative trial and signed written consent forms. None of the patients refused to participate. All patients undergoing an IVF treatment during this period, including oocyte recipients, were recruited in the study. Exclusion criteria were limited to cycles without good-quality supernumerary embryos and cycles from our special programs as infected patients (HIV, hepatitis B or C) and Preimplantation Genetic Diagnosis. Embryos were scored according to our grading system on the day of embryo transfer [31] and good-quality supernumerary embryos were allocated to one of the three groups for cryopreservation. A good-quality embryo is defined as timely cleaved (day 2: 4–6 cell; day 3: 8–12 cells) and with equal-size and shaped blastomeres and/or <30 % of fragmentation.

During the study period, a total of 1798 supernumerary good-quality day 2 or day 3 cleavage stage embryos from 645 fresh IVF cycles of 568 patients (mean age 33.4 years ± 5.2), according to a previously established weekly basis, were allocated to three cryopreservation groups: vitrification with the Irvine® procedure and media (Irvine group), vitrification with the Vitrolife® procedure and media (Vitrolife group) and slow-freezing procedure and media (slow-freezing group). The storage was performed using the Vitrification High Security kits and the High Security straws from CryoBioSystem® for vitrification and for slow-freezing groups, respectively.

The mean age of patients in the cryopreservation cycles (Irvine group: 33.7 years ± 5.4; Vitrolife group: 33.6 years ± 5.2; slow-freezing group: 33.1 years ± 5.0) was similar in all groups. The mean age of the donors (Irvine group: 31.0 years ± 4.2; Vitrolife group: 31.9 years ± 3.5; slow-freezing group: 31.4 years ± 5.3) and the proportion of cycles with frozen embryos obtained from oocyte donation was similar between the three studied groups (Irvine group: 12/255; Vitrolife group: 16/261; slow-freezing group: 18/260).

At the time of analysis (October 2012), 1055 of these embryos (58.6 %) were warmed for transfer in 776 initiated warming cycles of 420 patients (mean age 34.1 ± 5.2). Patient characteristics in the warming groups were similars. Of the all embryos included in the data analysed, 913/1055 were at day 2 cleavage stage and 142/1055 were at day 3 cleavage stage and the proportion of day 3 embryos was similar between the three different groups (Irvine: 45/330; Vitrolife: 37/330; slow-freezing: 60/395). Among all embryos warmed, 915/1055 were at 4–6 cells at cooling, only 16/1055 were at 2 cells and 124/1055 were at 8–12 cells. The efficacy of the three cryopreservation methods was assessed on these 1055 warmed embryos in terms of embryo survival and cleavage rates and in terms of implantation and live birth rates.

### Stimulation protocol for IVF

GnRH agonist and antagonist protocols were both used for controlled ovarian stimulation with urinary gonadotropins (hMG: Menopur®, Ferring, Switzerland) or recombinant FSH (Gonal F®, Merck-Serono, Switzerland or Puregon®, MSD, USA). Pituitary desensitization was obtained either using gonadotropin-releasing hormone agonists (Buserelin acetate: Suprefact® spray, Hoechst Inc., Germany or Triptorelin: Decapeptyl®, Ipsen, France) in short and long protocols or GnRH antagonist (Cetrorelix: Cetrotide®, Merck-Serono, Switzerland or Ganirelix: Orgalutran®, MSD, USA) started after 5 days of ovarian stimulation. When at least 3 follicles reached 17 mm in diameter, ovulation was induced by the administration of urinary human chorionic gonadotropin (hCG: Pregnyl®, MSD, USA) or recombinant hCG (Ovitrelle®, Merck-Serono, Switzerland). Oocyte retrieval was performed through vaginal puncture under ultrasound guidance 36 h later. Oocyte collection and laboratory procedures are described elsewhere [32].

For the warming cycles, patients underwent endometrial preparation for embryo transfers with either natural cycle or hormone therapy cycle (estradiol valerate: Progynova®, Schering, Belgium). The luteal phase was supported with micronized progesterone (Utrogestan®, Besins-Iscovesco, France) in separate doses of 2 × 200 mg in natural cycle and 3 × 200 mg in hormone cycle.

### Vitrification methods

#### Irvine vitrification procedure and media (Irvine group)

The technique of vitrification was performed according to the manufacturer’s instructions at room temperature. Embryos were placed into the Equilibration Solution (ES) for 5 min and then into the Vitrification Solution (VS) for 90 s. These solutions were used in sequence according to the step-wise microdrop vitrification protocol as described in the commercial kit instructions. ES is a HEPES buffered solution of Medium-199 containing gentamicin sulfate (35 μg/mL), 7.5 % (v/v) of each DMSO and ethylene glycol and 20 % (v/v) Dextran Serum Supplement. VS is a HEPES buffered solution of Medium-199 containing gentamicin sulfate (35 μg/mL), 15 % (v/v) of each DMSO and ethylene glycol, 20 % (v/v) Dextran Serum Supplement and 0.5 M sucrose. The embryos were loaded in high security straws (Vitrification High Security kits from CryoBioSystem) in a volume of maximum 1 μl. Straws were quickly plunged into Liquid Nitrogen (LN_2_) vertically and gently stirred in LN_2_ for a few seconds, then placed into the submerged LN_2_ filled cryotube into the cryo-freezer for long-term storage.

#### Irvine warming procedure and media

The technique of warming was performed according to the manufacturer’s instructions. Straws were removed from the cryotube and dipped into the Thawing Solution (TS) for 1 min at 37 °C. TS is a HEPES buffered solution of Medium-199 containing gentamicin sulfate (35 μg/mL), 1.0 M sucrose and 20 % (v/v) Dextran Serum Supplement. Embryos were placed successively into the Dilution Solution (DS) for 4 min and the washing solution (WS) for 9 min at room temperature. DS is a HEPES buffered solution of Medium-199 containing gentamicin sulfate (35 μg/mL), 0.5 M sucrose and 20 % (v/v) Dextran Serum Supplement. WS is a HEPES buffered solution of Medium-199 containing gentamicin sulfate (35 μg/mL) and 20 % (v/v) Dextran Serum Supplement. Finally, embryos were transferred to a pre-equilibrated culture dish containing microdrops of cleavage medium (day 2 cleavage stage) or blastocyst medium (day 3 cleavage stage) (Cook, Australia).

#### Vitrolife vitrification procedure and media (Vitrolife group)

The entire procedure was performed at 37 °C according to the manufacturer’s instructions. Embryos were placed into the Vitri 1™ Cleave Solution for 5 min, then into the Vitri 2™ Cleave Solution for 2 min, and finally into the Vitri 3™ Cleave Solution for 30 s.

The three solutions consist of a MOPS buffered medium containing gentamicin and human serum albumin. Vitri 1™ Cleave doesn’t contain any cryoprotectants, Vitri 2™ Cleave contains ethylene glycol as a cryoprotectant and Vitri 3™ Cleave contains ethylene glycol, propanediol, ficoll and sucrose as cryoprotectants. Cryoprotectants concentration is not detailed in the commercial kits. The embryos were loaded in high security straws (Vitrification High Security kits, CryoBioSystem) in a volume of maximum 1 μl. Straws were quickly plunged into Liquid Nitrogen (LN_2_) vertically and gently stirred in LN_2_ for a few seconds, then placed into the submerged LN_2_ filled cryotube into the cryo-freezer for long-term storage.

#### Vitrolife warming procedure and media

The technique of warming was performed according to the manufacturer’s instructions using the RapidWarm™ Cleave kit consisting of four different warming solutions. The entire procedure was performed at 37 °C. Straws were removed from the cryotube and dipped into the Warm1™ solution for 10 to 30 s, then into the Warm2™ solution for 1 min, after that into the Warm3™ solution for 2 min and finally into the Warm4™ solution for 5 min. The solutions consist of a MOPS buffered medium containing gentamicin and human serum albumin. Warm 1™ Cleave, Warm 2™ Cleave, Warm 3™ Cleave contain sucrose as a cryoprotectant while Warm 4™ Cleave contains no cryoprotectants. Cryoprotectants concentration is not detailed in the commercial kits. Finally, embryos were transferred to a pre-equilibrated culture dish containing microdrops of cleavage medium (day 2 cleavage stage) or blastocyst medium (day 3 cleavage stage) (Cook, Australia)

### Slow-freezing method

#### Freezing procedure and media (slow-freezing group)

The entire procedure was performed at room temperature. Embryos were placed for 10 min into a home-made freezing solution consisting of a manufactured buffered medium (Lonza, Belgium) containing gentamicin (40 μg/mL) and 0.5 % human serum albumin (CAF-DCF, Belgium) supplemented with 1.5 M 1,2-propanediol (PROH) and 0.1 M sucrose (Sigma–Aldrich S.r.L., UK). Embryos were then loaded into 0.3 ml high security straws (High Security straws, CryoBioSystem) and placed into the embryo freezer machine (BD1, Biotronics, UK). The programmed cooling curve was: from 25 °C to −7 °C (rate −2 °C/min), 15 min at −7 °C, seeding, 15 min at −7 °C, from −7 °C to −35 °C (rate −0.3 °C/min), from −35 °C to −80 °C (rate −45 °C/min). Finally straws were placed into the submerged LN_2_ filled cryotube into the cryo-freezer for long-term storage.

#### Thawing procedure and media

Straws were removed from the cryotube and, after 30 s at room temperature, plunged into a water bath for 5 s at 30 °C. Embryos were released into a home-made thawing solution consisting of buffered medium (Lonza, Belgium) containing gentamicin (40 μg/mL) and 0.5 % human serum albumin (CAF-DCF, Belgium) supplemented with 0.5 M sucrose (Sigma–Aldrich S.r.L., UK). Finally, embryos were transferred to a pre-equilibrated culture dish containing microdrops of cleavage medium (day 2 cleavage stage) or blastocyst medium (day 3 cleavage stage) (Cook, Australia).

### Embryo survival assessment and handling

Embryos were warmed the day before transfer, and were considered to have survived if they had 50 % or more surviving cells, evaluated as the absence of overt individual blastomeres degeneration morphologically visualized as necrotic cells. Embryos were considered fully intact only if they have 100 % surviving cells. Surviving embryos were put overnight in culture and only embryos that had cleaved were transferred. Usually, single embryo transfer was performed except in low prognosis cases were double transfer was allowed on patient request. The mean number of embryos transferred (Irvine 1.2 ± 0.4; Vitrolife group 1.2 ± 0.5; slow-freezing group 1.1 ± 0.4) was similar between the three different groups. The embryo survival rate was calculated as the percentage of embryos which survived after warming among the total number of embryos warmed. The embryo cleavage rate was calculated as the percentage of embryos which had cleaved among the surviving embryos. Only clinical pregnancies certified by the presence of at least one gestational sac at ultrasound were taken into account for calculation of implantation rate per transferred embryo as well as per warmed embryo. Live birth rates were calculated as the percentage of live birth per transferred embryo and per warmed embryo.

### Statistics

Data were compared with *χ* ^2^-tests using StatCalc software. Yates corrected p-values <0.05 were considered as being significant.

## Results

Results from the 1055 warmed embryos are summarised in Table 1. Post-warming survival of embryos in both vitrification methods were significantly higher compared to slow-freezing (*p* < 0.001). The proportion of embryos with 100 % intact blastomeres after warming was significantly higher after vitrification than after slow-freezing (*p* < 0.001). The cleavage rates of survived embryos after warming were significantly higher with vitrification methods (Irvine: *p* < 0.001; Vitrolife: *p* < 0.05 respectively) compared to slow-freezing. A significant difference in cleavage rate was observed between the two vitrification methods (*p* < 0.05) (Table 1).

**Table 1:** Summarised results of cryopreserved/warmed embryos by vitrification Irvine, vitrification Vitrolife and slow-freezing methods (*IR* implantation rate; *LB* live birth rate)

	Vitrification Irvine	Vitrification Vitrolife	Slow-freezing	Total
Cryopreserved embryos	575	524	699	1798
Warmed embryos	330	330	395	1055
Survived embryos (%)	295/330 (89.4 %)^a^	289/330 (87.6 %)^b^	252/395 (63.8 %)^a, b^	836/1055 (79.2 %)
Fully intact embryos (%)	267/330 (80.1 %)^a^	251/330 (76.1 %)^b^	175/395 (44.3 %)^a, b^	693/1055 (65.7 %)
Cleaved embryos (%)	259/295 (87.8 %)^a, c^	235/289 (81.3 %)^c, d^	182/252 (72.2 %)^a, d^	676/836 (80.9 %)
Transferred embryos (%)	259/330 (78.5 %)^a, c^	235/330 (71.2 %)^b, c^	182/395 (46.1 %)^a, b^	676/1055 (64.1 %)
IR/transferred embryo (%)	41/259 (15.8 %)	40/235 (17 %)	39/182 (21.4 %)	120/676 (17.7 %)
IR/warmed embryo (%)	41/330 (12.4 %)	40/330 (12.1 %)	39/395 (9.8 %)	120/1055 (11.3 %)
LB/transferred embryo (%)	26/259 (10 %)	25/235 (10.6 %)	24/182 (13.2 %)	75/676 (11.1 %)
LB/warmed embryo (%)	26/330 (7.8 %)	25/330 (7.5 %)	24/395 (6.1 %)	75/1055 (7.1 %)

Data were compared between groups. *P*-values: a vs a <0.001; b vs b <0.001; c vs c <0.05; d vs d <0.05)

Due to the higher post-warming survival and cleavage rate of embryos, significantly more transfers per warming cycle were performed when embryos were cryopreserved by both vitrification methods compared to slow-freezing method (*p* < 0.001). As summarised in Table 2, significant difference in the transfer rates per warming cycle was observed between the two vitrification methods (*p* < 0.05) (Table 2).

**Table 2:** Transfer rates per initiated warming cycle when embryos were cryopreserved by vitrification Irvine, vitrification Vitrolife and slow-freezing methods

	Vitrification Irvine	Vitrification Vitrolife	Slow-freezing
Initiated warming cycles	255	261	260
Transfer rate per initiated warming cycle	86.3 % ^a,c^	77.4 % ^b,c^	61.1 % ^a, b^
Mean number of embryos transferred	1.2 ± 0.4	1.2 ± 0.5	1.1 ± 0.4

Data were compared between groups. P-values: a vs a <0.001; b vs b <0.001; c vs c <0.05)

Implantation rate (IR) per embryo transferred and per embryo warmed and live birth (LB) per transferred embryo and per warmed embryo were no significant different (NS) between the three cryopreservation methods in the results obtained on the study sample (Table 1).

## Discussion

The slow-freezing method used for cryopreservtaion of human embryos has been largely used for cryopreservation of human embryos and recent improved protocols have optimised results [24, 25]. The vitrification is an increasingly popular method, based on an extreme viscosity of cryoprotectant solutions and a high cooling rate. To achieve the solidification of the solution below the glass transition temperature, high concentrations of cryoprotectants are used [33] and samples are loaded in a small volume. Different devices have been developed to achieve high cooling rate minimizing the exposure time to the vitrification solution [34–37]. If the exposure to cryoprotectants is too long, embryos suffer from toxicity of the solution [16–20]. For successful cryopreservation, an optimal balance between the cryoprotectant concentration and the cooling rate has to be established, to minimize toxic and osmotic injuries [38]. With recent improvements in media, devices and protocols, vitrification has become a reliable method. It is very simple and cheap; it can lead to high survival rates and good clinical outcomes [39]. One of the remaining concerns regarding vitrification is the possible toxic effect of the different cryoprotectant required at high concentration [40].

### Technical outcomes of different methods for cryopreservation of cleavage-stage human embryos

Results in survival and developmental rates as well as metabolic analysis of embryos obtained with different methods were evaluated and reported in the literature. In the study of Rama Raju et al. (2005) [28], cleavage stage embryos were vitrified using opened devices or conventional slow-freezing protocol. Post-thaw survival rate was significantly higher for the vitrification group compared to the slow-freezing group (*p* < 0.05). Later, Balaban et al. (2008) [29] cryopreserved cleavage stage embryos, donated for research by either conventional slow-freezing or vitrification using opened devises. Significantly more embryos survived after vitrification compared to slow-freezing (*p* < 0.05) and the progression rate to the blastocyst stage was significantly higher after vitrification than after slow-freezing (*p* = 0.02). Furthermore, metabolic analysis such as pyruvate uptake showed also a higher metabolic rate, leading to greater developmental rate to the blastocyst in the vitrification group. In a retrospective study, Rezazadeh Valojerdi et al. (2009) [30] evaluated the efficacy of vitrification using opened and slow-freezing for the cryopreservation of human cleavage stage embryos cryopreserved either with vitrification or slow-freezing. The survival rate and the post-warmed embryo morphology with all blastomeres intact were higher in the vitrification compared to the slow-freezing group confirming a minimal deleterious effect of vitrification on post-warming embryo morphology. In the study of Kartberg et al. (2008) [40], the authors evaluated the effect of cryoprotectants reporting compromised developmental ability if the cleavage stage embryos were vitrified when exposed to cryoprotectants for too long. Recently, Cobo et al. (2012) [41] assessed the embryo developmental and quality in a large study including almost 4.000 embryos when cryopreserved at different developmental stages and vitrified using open devices. Regarding early cleavage embryos, after warming, a very high number (~95 %) exhibited 100 % intact blastomeres after vitrification. In these studies, the embryos were vitrified using differents protocols but always by direct contact with liquid nitrogen. Even if there is no evidence in the published reports about contamination of cryopreserved embryos by direct contact with liquid nitrogen, devices such as Vitrification High Security kits from CryoBioSystem have been designed to reduce potential viral cross-contamination during storage in liquid nitrogen [42–45].

In our study, 1798 supernumerary good-quality cleavage embryos were cryopreserved by two different vitrification methods (DMSO-containing vs. DMSO-free vitrification solutions) or slow-freezing and the storage in all groups was performed in high security devices. In agreement with previous studies, results obtained on 1055 embryos showed that the survival rate significantly higher in the vitrification groups than in the slow-freezing group (*p* < 0.001). The proportion of embryos with 100 % intact blastomeres, and the cleavage rate was significantly higher in the vitrification groups than in the slow-freezing group (*p* < 0.001). Significant difference in cleavage rate was observed between the two vitrification methods (*p* < 0.05), which has never been reported in a randomised clinical trial, suggesting that Irvine method better preserve the embryo developmental competence after warming.

### Clinical outcomes of different methods for cryopreservation of cleavage-stage human embryos

Results in implantation and live birth rates obtained with different methods were evaluated and reported in the literature. Rama Raju et al. (2005) showed a higher IR in vitrification group compared to slow-freezing group of patients [28]. Later, Balaban et al. (2008) [29] confirmed a high clinical implantation rate after transfer of vitrified-warmed embryos. Rezazadeh Valojerdi et al. (2009) [30] also compared clinical outcomes after transfer of vitrified and slow-freezed embryos. In these studies patients were younger than in our study and 3 to 4 embryos cooled at day 3 with the best morphology were selected at warming and cultured for transfer. The implantation rate was higher in the vitrification group compared to slow-freezing group but the incidence of multiple pregnancy rate was also high. In the large study of Cobo et al. (2012) [41], the number of embryos to be transferred varied with each case, but typically 2 embryos were transferred on day 2 or day 3 and elective SET was considered only for blastocyst stage. The implantation rate of cleavage stage embryos was very high (27.2 % and 34.6 % for day 2 and day 3 respectively). As reported in these studies the clinical outcomes of the vitrification seems to be highly variable according to the centers [46]. The differences in implantation rate observed in the literature can be explained by many factors as the embryo selection for freezing or the patient characteristics. It may also be related to two major factors: the number of embryos warmed per cycle giving or not the possibility to choose the surviving embryos for transfer, and the use of different devices.

In our study, mostly SET were performed to reduce the risk of multiple pregnancy and closed devices were used to reduce the risk for cross contamination, with the disadvantage to slower vitrification speed compared to open devices [37–39, 43]. It may explain our lower clinical results compared to previous large studies [41]. The implantation rate (IR) and live birth (LB) rates per transferred embryo obtained were not significantly different (NS) between the three cryopreservation methods. Nevertheless, as the slow-freezing method was less efficient regarding the embryo survival and the fully intact survival rates, significantly more transfers were performed when embryos were cryopreserved by vitrification than by slow-freezing method. Based on the differences observed in our results, a statistical power calculation for the study size assessed the number of embryos needed at more than 2500 to obtain significant difference for the clinical outcomes.

### Summarized results and new findings of our study

In conclusion, this study provides evidence that vitrification, even in closed devices, is more efficient than slow-freezing cryopreservation in terms of survival and developmental rates of human cleavage embryos. Regardless of cryoprotectants and their concentration, vitrification gives better embryo survival rate compared to slow-freezing. Results observed between vitrification methods, suggest that Irvine method better preserve the embryo developmental competence after warming.

Even if no significant differences in implantation rate and live birth were observed between the three cryopreservation methods, through a higher survival rate and quality of the embryos at warming, vitrification may improve the clinical outcome of IVF by maximizing the cumulative efficiency of the cycle.
